# Development and Introduction of a Comprehensive Tobacco Control Policy in a Canadian Regional Health Authority

**Published:** 2007-03-15

**Authors:** Charl Els, Diane Kunyk, Gerry Predy, Mary Haase

**Affiliations:** Physicians for a Smoke-Free Canada and University of Alberta; University of Alberta and Physicians for a Smoke-Free Canada; Capital Health; Grant MacEwan College

## Abstract

**Background:**

Tobacco control policies in health care settings are necessary to protect patients, employees, physicians, visitors, and volunteers from the dangers of secondhand smoke. This report documents the process of developing and introducing a comprehensive tobacco control policy in one Canadian regional health authority.

**Context:**

Capital Health (CH), a health authority that has 30,000 employees and serves 1.6 million people, is responsible for 18 hospitals and primary care facilities, 33 continuing care facilities, 29 public health locations, and 9 community care facilities. CH recently determined that it needed to revise its tobacco control policy because its facilities had different directives regarding tobacco use, some of which did not reflect the best current knowledge about the health risks associated with exposure to secondhand smoke.

**Methods:**

The new smoke-free policy needed to be developed and executed within a narrow time frame, which required careful planning as well as the support of patients and CH staff members. An essential part of the new policy was the prevention of nicotine withdrawal among people required to undergo involuntary tobacco abstinence. The plan also included an integrated screening, intervention, and referral process designed to optimize health benefits for patients and staff members who smoked, as well as for those who did not.

**Consequences:**

CH decided to close all smoking rooms (including those in psychiatry, palliative care, geriatrics, eating disorder, and tuberculosis units), to ban smoking in outdoor areas, to stop all sales of tobacco products in CH facilities, to require smoke-free environments during home visitations, and to reject funding from the tobacco industry.

**Interpretation:**

By implementing a consistent ban on indoor and outdoor smoking, CH is contributing to a comprehensive tobacco control policy that is arguably a regional health authority's most profound opportunity for health promotion.

## Background

For decades, the use of tobacco products was tolerated, sometimes even in hospital settings. Some facilities even offered patients access to discounted tobacco products. Some health professionals still believe that special patient subgroups, such as those in residential mental health settings, cannot tolerate smoke-free policies (1). However, the anecdotal evidence upon which this belief is based (e.g., the effectiveness of tobacco products used as rewards for desired behavior and of therapists smoking with their patients while conducting therapy) has been largely discredited (1), and smoking rooms are no longer believed capable of adequately protecting people elsewhere in a facility from the harmful effects of secondhand smoke (SHS) (2). Furthermore, we now know that health care professionals face health risks from SHS exposure as the result of making in-home visits to patients who smoke (3).

Today we know that there is no risk-free level of SHS (2). Lenient tobacco control policies at health care facilities expose patients, staff, visitors, physicians, and volunteers to the harmful effects of SHS. In addition, such policies encourage patients who smoke to continue doing so, as well as possibly encourage nonsmokers to start or ex-smokers to relapse (4). Separating smokers from nonsmokers, cleaning the air, and ventilating buildings cannot eliminate all SHS exposure; only eliminating smoking in indoor spaces fully protects nonsmokers from exposure (2).

Current evidence suggests that a comprehensive tobacco control policy in health care settings, including asking patients to abstain from smoking while receiving care in their home, is necessary to ensure that health care professionals work in environments free of the risks associated with SHS exposure (5). Other aspects of a comprehensive tobacco control policy include instituting outdoor smoking bans, which makes it easier for health care facilities to enforce indoor smoke-free policies, encourage abstinence, reduce the risk for relapse among ex-smokers, and reduce the level of smoking among continuing tobacco users (6-9); eliminating tobacco sales, which prevents patients from accessing tobacco products while in the hospital; and rejecting tobacco industry funding, which not only ensures that health care organizations do not benefit financially from tobacco products but makes messages to patients about the health risks of tobacco exposure more credible and contributes to the denormalization of the tobacco industry. 

This report describes the process by which a comprehensive tobacco control policy was developed and introduced by Capital Health, one of nine regional health authorities in the province of Alberta. Canadian regional health authorities are provincial government entities responsible for hospitals, continuing care facilities, community health services, and public health programs within a defined region of a province.

## Context

Capital Health (CH) serves 1.6 million residents of the Edmonton area and is responsible for 18 hospitals and primary care facilities, 33 continuing care facilities, 29 public health programs, and 9 community care facilities. Because it recently added mental health services and had an increase in the territory it covers, CH came to be responsible for facilities with an array of different smoking policies. One of the mental health facilities integrated into CH actually sold tobacco products to patients. Although health care professionals engaged in selling these products have been called "secondary vectors" of tobacco dependence (4), the profits from tobacco sales at this facility created a financial dependence on tobacco money and subsequent resistance to policy change. Despite widespread knowledge about the dangers of smoking, CH approved significant expenditures to improve a designated smoking room in one of the CH facilities that allowed smoking only 3 months prior to a citywide ban on smoking that Edmonton instituted on July 1, 2005.

After that date, hospital units that continued to provide smoking rooms would have been subject to fines, as well as loss of credibility in the community. At about the same time, other health care sites in the region were also revising their tobacco control policies. For example, in May 2005, Alberta Hospital Edmonton, a large psychiatric facility, closed all of its designated smoking rooms, and on July 13, 2005, the Royal Alexandra Hospital became the first site to introduce a smoke-free policy on all outdoor hospital property. In late June 2005, the chief executive officer of CH announced the beginning of a process that would lead toward a comprehensive smoke-free policy for the entire health region.

Health care providers are also endangered by SHS exposure while caring for patients in their homes. An unpublished 2002 CH survey revealed that almost two thirds of community health nurses in CH were exposed to SHS while providing care in their clients' homes. The provision of home visitations by other types of service providers, including those offering environmental health, home care, and mental health services, suggests that SHS exposure in patients' homes may constitute an occupational health issue for a broad segment of health workers.

## Methods

The process of implementing a smoke-free policy was multifaceted and required careful planning and monitoring (1,4,9). The first steps were the creation of a regional committee to provide overall direction and the establishment of site committees to guide local activities. Although CH had announced that all facilities under its jurisdiction, including outside property and parking lots, would become smoke-free effective October 3, 2005, the regional committee decided that the special needs of some sensitive groups (including patients in mental health, brain injury, tuberculosis, and palliative care settings) required a phased-in approach over a further 6 months. CH's new smoke-free policy also included clauses that prohibited CH from accepting tobacco industry funds, insisted on the eventual closure of all CH smoking rooms, eliminated all tobacco sales at CH facilities, prohibited smoking on outdoor CH property, and required patients to abstain from smoking when receiving in-home care from CH employees.

CH sought legal opinions on three issues that related to its new tobacco policy: whether CH would be liable for injuries to patients leaving CH property in order to smoke; whether long-term patients have a right to smoke in a hospital when that hospital is considered to be their home; and whether forensic and certified patients can be prevented from leaving CH property to smoke (10). Opinions on all three issues offered support for the new policy. In Ontario, a psychiatric hospital's smoke-free policy was recently challenged, and the court in that case determined that patients did not have a constitutional right to smoke (11).

CH developed and implemented a communications plan to inform staff, physicians, visitors, patients, volunteers, contractors, and neighbors about the policy change. The plan included messages for specific target audiences, such as physicians and those receiving services in their home. Methods of communication included letters, advertisements through all media, signage, and CH Intranet and Internet messages.

The purpose of CH's smoke-free policy is not to enforce smoking cessation, but rather to protect everyone in CH facilities from SHS exposure. To minimize discomfort among tobacco users, CH attempted to prevent or minimize nicotine withdrawal among patients required to undergo involuntary abstinence while on CH property or while receiving care in their home (1,12). Study results suggest that the opportunity to experience abstinence from tobacco in a supportive environment, particularly without the discomfort of nicotine withdrawal, may encourage smokers to try to stop smoking altogether (6,8).

To prevent tobacco users from experiencing nicotine withdrawal while in a smoke-free environment, health care facilities need to institute screening to identify tobacco users; provide users with a supportive environment during their involuntary abstinence; and give them repeated opportunities to receive nicotine replacement therapy (NRT) in appropriate delivery routes, in sufficient dosages, and for adequate durations. To help patients avoid nicotine withdrawal, CH created a system whereby patients can receive NRT (a single type of therapy or a combination) and/or buproprion, an antidepressant often used as a smoking-cessation aid. CH received permission from Karl Fagertröm use an internationally validated rating scale to assess patients' nicotine dependence (13), asked for a region-wide hospital formulary change making the full spectrum of NRT products available to patients who need them, and recommended that NRT products be made available as ward stock for use by patients identified as smokers of more than 10 cigarettes per day (13). At the request of psychiatrists, CH also developed a medical directive for patients who become agitated because of nicotine withdrawal.

CH consulted with health care providers at units with existing smoking rooms (e.g., brain injury, rehabilitative care, and tuberculosis units) and formed committees to address the concerns of those providing services to palliative care, inpatient psychiatry, and home visitation patients. The following are examples of some of the concerns raised by this group of providers:

- Psychiatric patients would leave the premises to purchase cigarettes.
- Psychiatric patients would prostitute themselves to get tobacco.
- Tuberculosis patients would infect others by going off property to smoke.
- Staff would appear heartless by preventing palliative patients from smoking.
- Tobacco abstinence would be difficult to manage among cognitively impaired patients.
- Psychiatric patients would not access care because of smoking restrictions.
- Agitation and disorganized behavior among patients not allowed to smoke would threaten the safety of staff members.

Despite these concerns, CH determined that providing patients with a smoke-free health care environment without cues to encourage smoking was appropriate so long as patients were offered adequate dosages and combinations of NRT and personalized smoking-cessation support (1,12).

CH adapted a template (4) for closing the designated smoking rooms (Appendix A) and distributed it to all concerned parties in CH. The template included an implementation timeline and descriptions of related staff training, communication strategies, how tobacco-reduction activities were to be incorporated into current services offered to patients, and cessation support for nicotine-dependent staff.

After CH managers, policy makers, and frontline health care professionals identified the need to educate staff about tobacco dependence, CH provided 17 learning sessions to frontline staff on the prevention of nicotine withdrawal and the fundamentals of reducing tobacco use. CH obtained permission from the Registered Nurses' Association of Ontario to implement its e-learning module and its Web-based manual *Integrating Tobacco Cessation into Daily Nursing Practice *(14). To ensure that their approach to instituting a smoke-free environment was consistent with approaches used elsewhere in Canada, CH officials also consulted with officials from the Centre for Addiction and Mental Health, the Mental Health Centre Penetanguishene, and other Canadian institutions.

Health Canada, Pfizer Consumer Healthcare, and the Alberta Tobacco Reduction Strategy supported the development of the Tobacco Reduction and Cessation Project (TRaC) (15), a joint project between the University of Alberta and Capital Health, with the objective of integrating tobacco reduction and cessation activities into current health care programs. TRaC helped train more than 200 CH staff members and enhanced the capacity of regional health care providers to integrate tobacco reduction and cessation into their programs through methods such as motivation enhancement therapy, cognitive behavioral therapy, and evidence-based NRT.

Capital Health's Occupational Health, Safety, and Wellness (OHS&W) program expanded its tobacco reduction services for employees, physicians, and their families by establishing a tobacco reduction and cessation clinic run by a certified addiction specialist and by providing an 80% subsidy on all smoking cessation aids, including buproprion. This expansion of services is considered a critical element in efforts to help CH staff members comply with CH's revision of its smoking policies.

## Consequences

The introduction of the new tobacco control directive followed evidence-based standards from several sources (5,10), including *Treating Tobacco Use and Dependence: A Clinical Practice Guideline* (12). Based on a broad review of scientific evidence, this publication of the U.S. Public Health Service provides recommendations for smoking-cessation treatment, as well as information on the effectiveness of various types of treatment. Because CH officials had less than 3 months to develop and introduce the new nonsmoking policy throughout all CH facilities, they were unable to conduct a formal evaluation; however, local and regional committees do provide continuous feedback concerning the effectiveness of the policy and any problems encountered by patients or staff.

To accommodate patients unable to tolerate involuntary tobacco abstinence, CH initiated measures to screen patients for tobacco consumption and negotiated access to the entire range of nicotine replacement products in Canada. As a result of these actions, all CH patients have access to nicotine gum (except in forensic settings) and the patch, and use of the nicotine inhaler is being tested at some sites**.** CH also instituted a policy that gives nurses the authority to treat hospital patients' involuntary withdrawal from tobacco if a physician is not immediately available.

Following evidence-based guidelines for the incorporation of tobacco dependence treatment into routine hospital and clinical care (12), CH provided extensive training for staff members during the first phase of TRaC (15,16); however, the skills that staff members developed through this project, including assessing the tobacco consumption of patients and hospital staff, preventing smokers from experiencing nicotine withdrawal, and instituting tobacco reduction and cessation interventions, have not been fully integrated by CH. A considerable investment will be required for tobacco abstinence and cessation measures to be instituted at all CH sites, a finding that echoes the experiences of health care facilities in other regions of Canada and in Australia (1).

Communication, consistency, coordination, and full administrative support are essential to the success of any major policy change (1,17). CH communicated its new nonsmoking policy through its use of regional and site committees, through the development and execution of its communications plan, through coordination and partnership with regional public relations organizations, and through communications with providers of community and inpatient services. CH also has identified a need for ongoing communication of its new policy.

To date, the CH policy change has resulted in no behavioral indicators of unrest or violence and no noticeable changes in the levels of psychopathology among patients at CH facilities. These findings are consistent with those from a review of studies conducted in the United States, Canada, and Australia (1). CH staff members no longer smoke with patients, and nurses no longer use smoking as a tool in the clinical management of patients. Despite minor violations of the new smoke-free policy during the early stages of its implementation and challenges in enforcing it at several sites, CH has found no compelling reasons to reverse the policy and now considers its smoke-free policy to have been safely and effectively implemented in all of its facilities (3).

## Interpretation

Comprehensive tobacco-control policies are necessary to protect the health of patients, employees, physicians, visitors, and volunteers. The implementation of CH's smoke-free policy affected almost every employee subgroup, including security, facilities management, public relations, physicians, management, and frontline staff. The policy was developed and implemented within a 3-month timeframe, with a short but adequate grace period to accommodate special subgroups.

A critical step in the development of CH's smoke-free policy was the decision that its primary purpose should be to prevent CH staff and patients from being exposed to SHS. CH then determined that the primary goal of any intervention offered as part of this policy should be to prevent tobacco consumers from experiencing nicotine withdrawal during involuntary abstinence and thereby support their compliance with the policy. CH's new policy offers patients and staff an environment without cues to encourage smoking, appropriate screening for tobacco use, and appropriate nicotine replacement products for those who need them. CH is also developing a system of support for personalized tobacco reduction and cessation efforts.

Aspects of its new tobacco control policy that CH still must improve include developing steps to incorporate the new standing orders allowing nurses to dispense NRT products at all CH units and facilities, further integrating tobacco reduction and cessation activities into standard CH operating procedures, providing ongoing tobacco control education to CH staff members, and managing possible consequences of the policy. Such consequences may include a need for more security staff to enforce the no-smoking directive, measures to keep patients or staff from injuring themselves when leaving the property to smoke during frigid winter weather, and potential legal challenges to the new policy. Once comprehensive screening, support measures, and cessation services are in place, CH will have less need to focus on providing increased security to support the no-smoking policy or on protecting smokers from the elements.

Smoke-free policies should be viewed as a part of a larger comprehensive tobacco-control strategy (5), the implementation of which is arguably the most profound opportunity for health promotion by a regional health authority. Furthermore, efforts to implement smoke-free policies at health care facilities, particularly efforts to support abstinence, can dovetail with larger, regional tobacco-control strategies (5,13).

## Appendix. Timeline for Closing Designated Smoking Rooms in Capital Health Facilities

**Table:**

**Planning Stage**	Ensure that the closure plan is endorsed by the Capital health (CH) corporate office and medical leaders. Inform CH staff members of the plan (ongoing). Offer cessation assistance to all new inpatients. Establish a steering committee at each site with a designated smoking room (DSR). Consult with facility staff and physicians to establish a protocol for smoking-cessation services. Estimate the prevalence of tobacco dependence among facility staff members. Disseminate the process by which staff members can access smoking-cessation assistance. Encourage staff members who smoke to engage in cessation efforts (ongoing). Inform patients of the dangers of smoking and of the imminent closure of DSRs. Establish the full range of nicotine replacement therapy (NRT) products on hospital formularies across the region. Coordinate proposed DSR closures with the tobacco committees at each affected hospital. Conduct a media campaign to inform the public of the imminent DSR closures. Cease spending on ventilation technology. Use grand rounds to inform to inform CH personnel throughout the region of the new no-smoking policy.
**Three months before closure**	Announce the closure process to patients and staff. Place visible reminders of the date that smoking rooms will close. Allow only patients with no off-ward privileges into designated smoking rooms (DSRs). Place clear tobacco-related health messages in all DSRs. Remove all smoking-related social cues from DSRs. Disseminate to all staff members information about the 5A approach (ask, advise, assess, assist, arrange), as well as about nicotine replacement therapy (NRT) and bupropion guidelines. Obtain permission from the Registered Nurses Association of Toronto to use a set of e-learning modules that it developed for nursing staff. Disseminate protocols of cessation interventions to all CH staff members. Initiate weekly cessation group sessions for patients on each ward. Increase the availability of smoking-cessation self-help materials for staff members and patients. Start monitoring NRT usage. Establish a consultation service that allows physicians and nurses to consult with a tobacco specialist on tobacco-related clinical matters. Close DSRs 25% of the time. Discontinue selling or dispensing tobacco products to patients. Implement standing orders for NRT. Provide a Ward Atmosphere Scale in all units. Obtain a legal opinion concerning the legality of closing the DSRs.
**Two months before**	Conduct staff focus groups at each site where a DSR is to be closed. Consult with a council representing patients. Provide written information about the impending no-smoking policy to all prospective inpatients. Discuss proposed changes with housekeeping, security, maintenance, and other departments and provide departments with updates at 2-week intervals. Close DSRs 50% of the time.
**One month before**	Close DSRs 75% of the time, and close them entirely during evening hours. Discuss the DSR closures with physicians at grand rounds.
**Three weeks before**	Continue gradually reducing the amount of time that DSRs are open (ongoing).
**Two weeks before**	Establish groups in which patients can vent their reactions about the no-smoking policy.
**One week before**	Conduct ward-level community meetings. Offer voluntary discharges to psychiatric patients unless their admission was involuntary.
**Closure of Designated Smoking Rooms**	
**One day after**	Clean former DSRs and if necessary repaint them. Inform staff members that the current "fragrance policy" barring them from wearing strong perfumes or colognes may be expanded to include tobacco odor.
**Seven days after**	Monitor violations of the no-smoking policy and refine protocols for enforcing it. Monitor patient complaints and discuss these complaints with patient representatives.
**One month after**	Collect formal feedback from staff regarding contraband, violations, patient reactions, and Ward Atmosphere Scale results.
**Two months after**	Implement a protocol for dealing with violations of the no-smoking policy.
**Three months after**	Begin conducting daily searches for contraband.
**Four months after**	Offer staff members refresher courses on cessation methods.
**Five months after**	Assess staff members' attitudes, skills, and knowledge concerning the tobacco ban and smoking-cessation methods. Reassess the prevalence of smoking among staff members. Begin ongoing education for staff members about accessing the effectiveness of smoking-cessation interventions.
**Six months after**	Conduct community meetings to discuss the no-smoking policy and implementation protocols and to find solutions to any problems that may have arisen. Conduct an occupational health and safety assessment to compare the occupational health status in affected hospitals before the DSR closures with the status after the closures. Review the amount of medication used to restrain patients before and after the DSR closures, as well as the number of patients using NRT and the number of critical incidents that threatened the safety of patients or staff members. Celebrate the facility's 6-month smoke-free status.
**Twelve months after**	Celebrate the first anniversary of the facility's smoke-free status. Plan an event and medical release to publicize the number of lives and health care dollars saved by the new policy. Administer the Ward Atmosphere Scale in wards across the region.
